# Increased nucleoside diphosphate kinase activity induces white spot syndrome virus infection in *Litopenaeus vannamei*

**DOI:** 10.1371/journal.pone.0175741

**Published:** 2017-05-15

**Authors:** Peng-fei Liu, Qing-hui Liu, Yin Wu, Jie Huang

**Affiliations:** 1 Key Laboratory of Sustainable Development of Marine Fisheries, Ministry of Agriculture, Yellow Sea Fisheries Research Institute, Chinese Academy of Fishery Sciences, Qingdao, China; 2 Dalian Ocean University, Dalian, China; 3 National Laboratory for Marine Science and Technology, Qingdao, China; Shanghai Ocean University, CHINA

## Abstract

Nucleoside diphosphate kinase (NDK), which has the same sequence as oncoprotein (OP) in humans, can induce nucleoside triphosphates in DNA replication by maintenance of the deoxynucleotide triphosphate (dNTP’s) and is known to be regulated by viral infection in the shrimp *Litopenaeus vannamei*. This paper describes the relationship between NDK and white spot syndrome virus (WSSV) infection. The recombinant NDK was produced by a prokaryotic expression system. WSSV copy numbers and mRNA levels of *IE1* and *VP28* were significantly increased in shrimp injected with recombinant NDK at 72 h after WSSV infection. After synthesizing dsRNA-NDK and confirming the efficacy of NDK silencing, we recorded the cumulative mortality of WSSV-infected shrimp injected with NDK and dsRNA-NDK. A comparison between the results demonstrated that silencing NDK delayed the death of shrimps. These findings indicate that NDK has an important role influencing the replication of WSSV replication in shrimp. Furthermore, NDK may have potential target as a new therapeutic strategy against WSSV infection in shrimp.

## Introduction

Nucleoside diphosphate kinase (NDK) is an essential enzyme which is required for the production of nucleoside triphosphates by a reaction that catalyses the transfer of a phosphoryl group from nucleoside 5′-triphosphates to nucleoside 5′-diphosphates, it also plays a key role in maintaining intracellular energy resources [1, 2]. Members of the *Nme* gene family encode nucleoside diphosphate kinases [3]. Recent research indicates that members of the nm23 family display multiple functions in diverse biological processes such as signal transduction, growth control, differentiation, cell migration, and the promotion of cancer [4–6].

A NDK cDNA (GenBank: DQ907945.1) containing 151 residues was found in the shrimp *Litopenaeus vannamei* during a transcriptome study [7]. The sequence of the shrimp NDK was highly conserved with 40 and 60% identity among eukaryotic and bacterial NDKs, respectively. On analyzing the amino acid sequence, we discovered that it was similar to the human oncoprotein nm23, and when shrimps were infected by white spot syndrome virus (WSSV), NDK was up-regulated [8]. However, the relevance of NDK to viral pathology and the underlying mechanisms are still largely unknown in crustaceans.

Among the known viral pathogens capable of infecting shrimp, WSSV is probably the most damaging and has induced huge economic losses in the shrimp industry worldwide since it was discovered in Taiwan in 1992 [9]. To date, there is no effective antiviral treatment to control the outbreaks and inhibit the prevalence of WSSV.

Most of the studies are available and indicate that NDK can act as an antiviral by targeting several pathogens to process the activation of nucleotide or nucleoside analogues. Therefore, the aim of this study is to validate functional interaction between NDK and WSSV by prokaryotic expression and RNA interference (RNAi). To identify the role of NDK in the process of WSSV infection in shrimps and the interactions that exist between them.

## Materials and methods

### Ethics statement

The use of non-human primates in research. All shrimps in this study were handled in strict accordance with China legislation on scientific procedures on living animals. The protocol was approved by the ethics committee at Dalian Ocean University.

### WSSV inoculum preparation and experimental shrimp collection and maintenance

Shrimp (*L*. *vannamei*), weighing 7.2–8.7 g and 8.9–10.5 cm in length, were collected from a larval production laboratory named Qingdao Baorong Aquatic Product Techonology CO., LTD in Qingdao, Shandong Province, China. They were acclimatized temporarily to laboratory conditions at a temperature of 23.0 to 24.5°C in cycling-filtered plastic tanks containing air stones to provide constant aeration for two weeks. Prior to experimental use, animals were fed twice daily with commercial shrimp feed. Otherwise, temperature, salinity and pH were recorded daily. WSSV inoculum was prepared from the infection of healthy crayfish (confirmed by PCR using qw primers, listed in Table 1) *Procambarus clarkii* as described previously [10]. Primers qw were synthesised according to our previous study [11]. The tissues of infected crayfish, excluding the hepatopancreas, were homogenized in TNE buffer (50 mM Tris–HCl, 400 mM NaCl, 5 mM EDTA, pH 8.5) and then centrifuged at 3500 × *g* for 5 min at 4°C. The supernatant was then centrifuged at 30, 000 × *g* for 30 min at 4°C after being filtered by nylon net (400 mesh, 30–1500 μm). Then, the upper loose pellet was rinsed carefully and the lower white pellet was suspended in 10 ml TN buffer (20 mM Tris–HCl, 400 mM NaCl, pH 7.4). After centrifugation at 3500 × *g* for 5 min, the virus particles were sedimented by centrifugation at 30, 000 × *g* for 20 min at 4°C, then resuspended and kept in 1 ml TN buffer. Under electron micrograph, the purified WSSV were observed and virions were calculated according to following formula: *C*(virions/μl) = 7.5 × 10^6^ μm^2^ × 50 × *N*/20 μm^2^ × 2 μl = 9.375 × 10^6^ × *N*/μl, where *C* denotes the viral concentration, as well as *N* denotes the mean number of the virus particles in the 20 images [12].

**Table 1:** Primers sequence.

Primer name	Forward/reverse sequence(5’-3’)	Accession number	Amplification	TM	Reaction efficiency
qW-f	CTCTTGTGGTTCATCAGGGGC	AF332093.1	1357 bp	61.0	99.6%
qW-r	CTGGATTTTCTCTCAGGGTCTTTAGT			60.3	
Actin-f	CATCAAGGAGAAACTGTGCT	XM_018157137	214 bp	55.7	99.8%
Actin-r	GATGGAGTTGTAGGTGGTCT			56.3	
NDK(s)	TACTCGAGATGGTTCGCGAACGCAC	ABI93176.1	471 bp	71.3	99.6%
NDK(a)	GCAAGCTTCGCTCGTAGATCCAGCT			69.4	
NDK-F	TTCGCGAACGCACTTTCATCGCC	ABI93176.1	440 bp	66.8	98.8%
NDK-T7-F	GATCACTAATACGACTCACTATAGGGTTCGCGAACGCACTTTCATCGCC			73.9	
NDK-R	TTGAACCACAGAGCAATCTCC			58.2	
NDK-T7-R	GATCACTAATACGACTCACTATAGGGTTGAACCACAGAGCAATCTCC			70.5	
EGFP-F	CAGTGCTTCAGCCGCTACCC	AAB02574.1	327 bp	63.5	98.3%
EGFP-T7-F	GATCACTAATACGACTCACTATAGGGCAGTGCTTCAGCCGCTACCC			73.8	
EGFP-R	AGTTCACCTTGATGCCGTTCTT			60.5	
EGFP-T7-R	GATCACTAATACGACTCACTATAGGGAGTTCACCTTGATGCCGTTCTT			71.2	
IE-f	TGGCACAACAACAGACCCTA	AAL88927.1	101 bp	59.2	99.1%
IE-r	CTTTCCTTGAAGTACGAGAC			53.4	
VP28-f	GGGAACATTCAAGATGTGGA	AAL88896.1	121 bp	55.3 60.0	99.3%
VP28-r	GGTGAAGGAGGAGGTGTTGG				
NDK-qF	TGAAGATCTCTTGAAGCAGC	ABI93176.1	203 bp	52.7	99.5%
NDK-qR	CTTCAATGCAGAAATCTCCG			55.8	

### Prokaryotic expression and purification of recombinant NDK in *Escherichia coli*

Recombinant NDK was amplified by PCR with forward primer NDK(s) and reverse primer NDK(r) (Table 1). The PCR product, which included the restriction sites *Hind*III and *Xho*I, was recovered through gel extraction using a kit (Zymo, Orange County, USA). Then the PCR product was cloned into the prokaryotic expression vector pBAD/gIIIA vector and transformed into *E*. *coli* TNDK 10 cells (TaKaRa, Dalian, China) by T4 DNA ligase (TaKaRa). We selected recombinant clones by ampicillin resistance and picked monoclones to culture in 10 mL of LB liquid culture medium overnight at 37°C with agitation. Plasmid DNA was extracted using an Extraction Kit (TIANGEN, Beijing, China) and digested with the restriction enzymes *Hind*III and *Xho*I. Finally, recombinant NDK (rNDK) was confirmed in the selected positive clone by PCR and sequencing. The cultures were incubated at 37°C for 5 h and 0.2% L-arabinose was added. The cells were centrifuged at 10,000 × *g* for 10 min at 4°C and resuspended in phosphate-buffered saline (PBS) (135 mM NaCl, 2.7 mM KCl, 1.5 mM KH_2_PO_4_, 8 mM K_2_PO_4_, pH 7.2). As the fusion protein had a His-tag, we collected the supernatant and added it to Ni-NTA resin for purification. Then the solution was filtered by 4 M guanidine hydrochloride, 2 M guanidine hydrochloride and no guanidine hydrochloride in turn. And each dialysis step was carried out for at least 12 h at 4°C. Combined with methods published in our previous research, we obtained purified rNDK and analyzed it by sodium dodecyl sulfate-polyacrylamide gel electrophoresis (SDS-PAGE) [13].

### Relative quantification of NDK gene expression in tissues

In order to investigate the mRNA levels of NDK gene expression in *L*. *vannamei*, six tissues including lymphoid, gill, hepatopancreas, muscle, intestine and hemocytes were extracted individually from three shrimp selected at random.

Total RNA was extracted from tissues and then reverse transcribed into cDNA by using RNAiso Plus (TaKaRa, Dalian, China) reagent. In order to assess the quality of the RNA, a NanoDrop 2000 spectrophotometer (NanoDrop Technologies, Wilmington, USA) was used to measure the absorbance of RNA at 260 and 280 nm, and 1.5% formaldehyde agarose gel electrophoresis was used to analyze the RNA integrity [14]. According to the manufacture’s protocol, PrimeScript RT reagent Kit with gDNA Eraser (TaKaRa) was used to synthesize the cDNA with 1 μg total RNA. And then all cDNAs were diluted to 50 ng/μl with diethylpyrocarbonate (DEPC)-treated H_2_O and restored at −20°C for reverse transcription PCR (RT-PCR). Primers of NDK called NDK-qF and NDK-qR were designed based on published *L*. *vannamei* cDNA sequences of *NDK* (Table 1). The qRT-PCR was performed in a 25 μL volume containing 12.5 μL of 2 × SYBR Premix Ex Taq (Roche, Beijing, China), 1 μL DNA, 10.5 μL DEPC H_2_O, 0.5 μL of each of the forward and reverse primers (1 μmol/L) and 1 μL cDNA template. The reaction conditions for qRT-PCR were carried out with initial denaturation at 94°C for 5 min followed by 40 cycles of 94°C for 30 s, annealing at 60°C for 30 s, extension at 72°C for 30 s and 72°C for 10 min. A melting curve was produced for each amplification product. The data obtained were statistically analyzed and calculated using the 2^-△△ct^ method [15] and given as means ± SD standard deviation. Differences in mortality were tested for statistical significance. Bio-Rad CFX Manager 3.0 software was used to analyze the qRT-PCR data.

### NDK effect on WSSV-challenged shrimp

#### Relative quantification of IE1, VP28 gene expression in gills

To analyze the function of NDK in *L*. *vannamei* after WSSV infection, 36 shrimps were chosen at random and divided into three groups which were pretreated with PBS, BSA (1 mg/mL), and NDK respectively. First, PBS (30 μL), BSA (30 μg), or NDK (30 μg) were injected into the third abdominal somite of shrimps using a 1 mL tuberculin syringe. After 2 h, shrimps in the NDK group and BSA group were challenged with 30 μL of purified WSSV (10^6^–10^7^ copies/μL) by intramuscular injection, while the control group was injected with 30 μL PBS. The infection dose was determined in our previous study [13], and PBS and BSA were filtered through a 0.22 μm membrane. During the WSSV challenge, gills from shrimps were collected at 0, 24, 48 and 72 h. Then, total RNA from tissues were reverse transcribed into cDNA by the same methods mentioned above. For qRT-PCR, specific primers of IE1 and VP28 were used and β-actin mRNA was used as a control (Table 1).

#### Quantitative assessment of the WSSV copies by qRT-PCR

Viral load was quantified by measuring the WSSV-copies in the gills of experimental shrimps as gills are highly active metabolic tissues in decapod crustaceans [16]. Based on the WSSV sequence (AF332093.1), primers qw were synthesized as our previous study described [11] to assess copy numbers of WSSV in shrimps. At 0, 24, 48, and 72 h, all samples were extracted from WSSV-infected and mock-infected shrimps’ gills by using a DNA extraction kit (TIANGEN, Beijing, China). DNA from all samples was extracted and used as template to quantify viral load by qPCR. Triplicate reactions of qPCR were performed in a BioRad ICycler real-time PCR system according to Durand and Lightner [17]. A 25 μL volume including 12.5 μL of 2 × SYBR Premix Ex Taq (Takara), 1 μL DNA, 10.5 μL DEPC H_2_O, 0.5 μL each of forward and reverse primers (1 μmol/L) and 1 μL DNA template. PCR conditions were denaturing at 94°C for 5 min followed by 40 cycles of 94°C for 30 min, annealing at 60°C for 30 s, and extension at 72°C for 30 s. A melting curve was produced for each amplification product. In this experiment, 10^4^–10^9^ (copies/μL) concentration gradient of plasmid containing WSSV-DNA were constructed as a standard curve to quantify the copy numbers of WSSV-infected samples. Dilution series of WSSV DNA were amplified with WSSV specific primers: forward primer qw-a and reverse primer qw-s (Table 1).

#### Cumulative mortality analysis

The shrimps were randomly divided into four groups including three parallel groups (*n* = 15 in each group) and injected with either NDK, BSA, WSSV or PBS by 1 mL tuberculin syringe. The NDK group and BSA group were injected with 30 μg NDK and 30 μg BSA, respectively. The method of injection was the same as in section 2.4.1. Two hours later, these two groups and the WSSV-infected group were injected with 30 μL of purified WSSV (10^6^–10^7^ copies/μL), while the PBS group was injected with 30 μL PBS. Mortality was monitored every 12 h and the cumulative mortality was recorded each day.

### Production of dsRNA-NDK

By using the kit of TranscriptAid T7 High Yield Transcription (Thermo, Beijing, China) according to the manufacturer’s instructions, dsRNA-NDK were constructed from a region of *L*. *vannamei* NDK (317 bp) (GenBank: DQ907945.1) amplified using primers NDK-T7-F, NDK-F NDK-T7-R and NDK-R (Table 1). The dsRNA-Enhanced Green Fluorescent Protein (EGFP) was amplified using primers EGFP-T7-F, EGFP-F, EGFP-T7-R and EGFP-R (Table 1). Briefly, DNA templates for the production of dsRNA-NDK and dsRNA-EGFP were amplified by PCR using gene-specific primers (Table 1) at the 5′ terminus to produce sense and antisense RNA strands separately. The PCR conditions were the same as previously described with annealing temperatures of 60°C and 59°C for dsRNA-NDK and dsRNA-EGFP, respectively. Then, the single-stranded RNA was annealed to generate dsRNA. After purification, the dsRNA-NDK and dsRNA-EGFP were quantified and then stored at −80°C [18–20].

### In vivo RNAi assay in Shrimp

#### Effective inhibition of dsRNA-NDK in hemocytes, lymphoid and hepatopancreas of shrimps

Expressed dsRNA-NDK (30 μg/shrimp), dsRNA-EGFP (30 μg/shrimp) and PBS (30 μL, control) were injected into shrimps that were divided into three groups to give 36 shrimps in total. At 0, 24, 48 and 72 h after injection, three shrimps were randomly selected and tissues of hemocytes, lymphoid and hepatopancreas were divided. Each total RNA sample was extracted, reverse transcribed and amplified in RT-qPCR to obtain NDK gene expression with the β-actin gene as a loading control.

#### Gene expression of *IE1* and *VP28* of WSSV-infected shrimp injected with expressed dsRNA-NDK in gills

dsRNA-NDK (30 μg/shrimp), dsRNA-EGFP (30 μg/shrimp) and 30 μL PBS were delivered into shrimps, and 30 μL WSSV (10^6^–10^7^ copies/μL) were injected after two days. Three gills were collected at 0, 24, 48 and 72 h from shrimps respectively. As above, we obtained the gene expression data of *IE1* and *VP28*.

#### Effects of dsRNA-NDK on the mortality of WSSV-infected shrimp

In order to test the capacity of dsRNA-NDK interference on WSSV, we chose 145 shrimps and randomly divided them into three groups including three parallel groups (*n* = 15 in each group). The experimental group was injected with dsRNA-NDK (30 μg/shrimp), whereas the control groups were injected with dsRNA-EGFP (30 μg/shrimp) and 30 μL PBS, respectively. After two days, the WSSV-infected group and these two control groups were injected with 30 μL of the purified WSSV (10^6^–10^7^ copies/μL). The cumulative mortality was recorded each day and the data were analyzed.

### Statistical analysis

The software randomly SPSS 18.0 was used to analysis the data. Data analyses were done by one-way analysis of variance (ANOVA) and Tukey’ s comparison of means. And all data were expressed as the means ± standard deviation. A probability value of less than 0.05 was considered significant.

## Results

### rNDK recombinant expression and mass spectrometry assay

**Fig 1:**
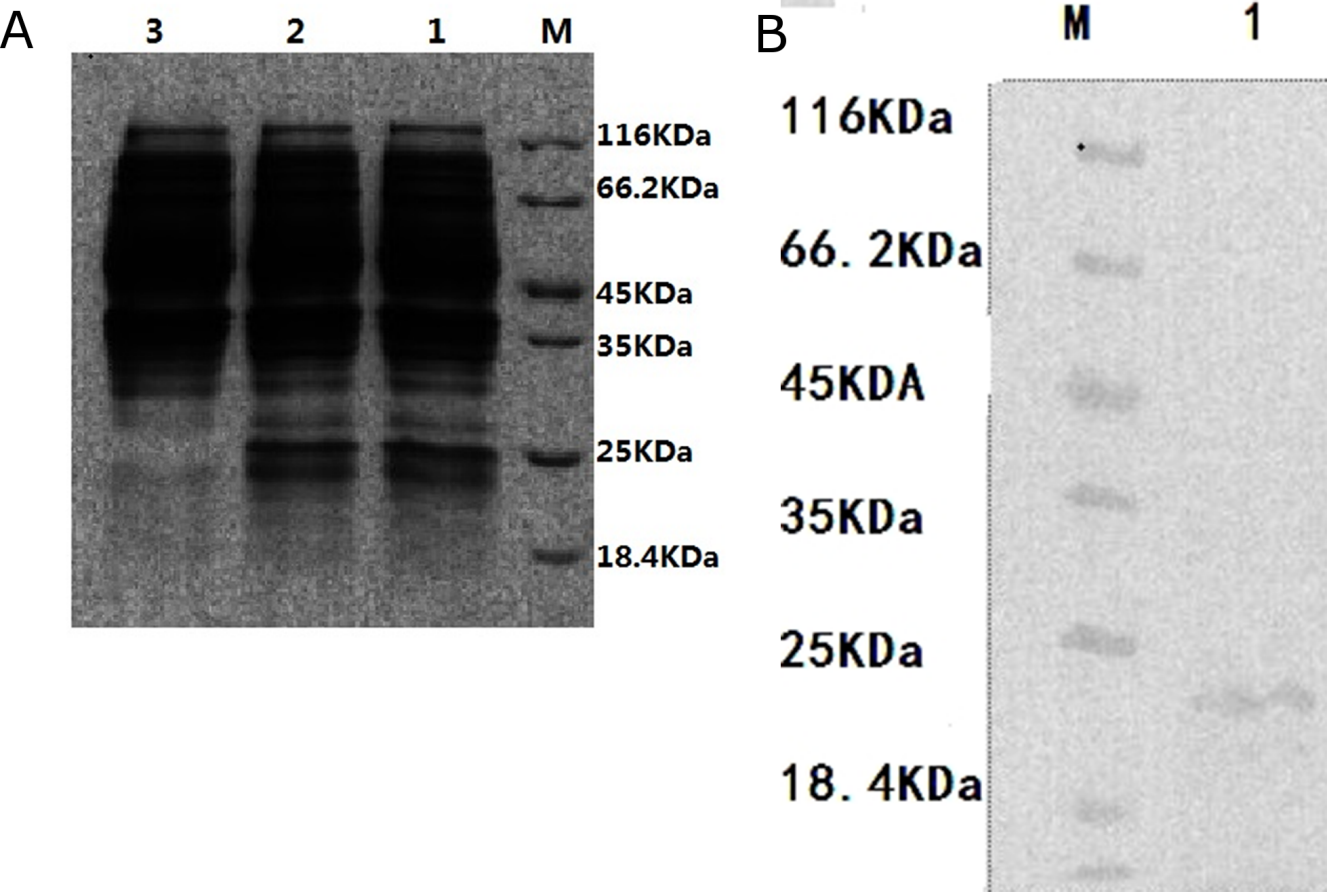
SDS–PAGE analysis of of NDK expressed from vector pBAD/gIII A. (A) SDS-PAGE analysis of induced and non-induced pBAD⁄gIII A-NDK-TNDK10 (containing NDK). Induced pBAD⁄gIII A-NDK-TNDK10 (*Lane* 1–2). Non-induced pBAD⁄gIII A-NDK-TNDK10 (*Lane* 3). M: protein marker (B) SDS-PAGE of expressed purified proteins NDK (*Lane* 1). M: protein marker

Through prokaryotic expression, recombinant NDK was successfully expressed in *E*. *coli*. From SDS-PAGE, the apparent molecular mass of rNDK was estimated to be 25 KDa (Fig 1). No protein band was found at this position in the non-induced *E*. *coli*.

To identify the protein, bands were cut from SDS-PAGE gels and identified by using MALDI-TOF-MS and analyzed by using the MS-FIT system. We observed that seven peptide fragments of the rNDK were matched with the deduced amino acid sequence of NDK from *L*. *vannamei* (Table 2). This indicates that the recombinant protein was NDK (Table 2).

**Table 2:** Measured and calculated molecular masses of tryptic peptides 25kD protein.

NDK		[shrimp white spot syndrome virus]		gi|20149139	
Start	End	Peptide	Observed	Mr(expt)	Mr (calc)
**18**	**25**	GLIGEIIK	842.47	841.46	841.53
**105**	**113**	GDFCIEVGR	995.42	994.41	994.45
**18**	**26**	GLIGEIIKR	998.59	997.58	997.63
**39**	**48**	YIQASEDLLK	1179.55	1178.54	1178.62
**6**	**17**	TFIAVKPDGVQR	1330.67	1329.66	1329.74
**114**	**127**	NIIHGSDSVESANK	1470.63	1469.63	1469.71
**88**	**104**	VMMGETRPADSKPGTIR	1845.81	1844.80	1844.92

Seven matched peptide fragments and their positions in the deduced amino acids of rNDK by electrospray ionization-mass spectrometry/mass spectrometry (ESI-MS/MS) analysis. Start-End: position of the sequence; MW: molecular weight.

### Shrimp NDK mRNA levels in tissues

**Fig 2:**
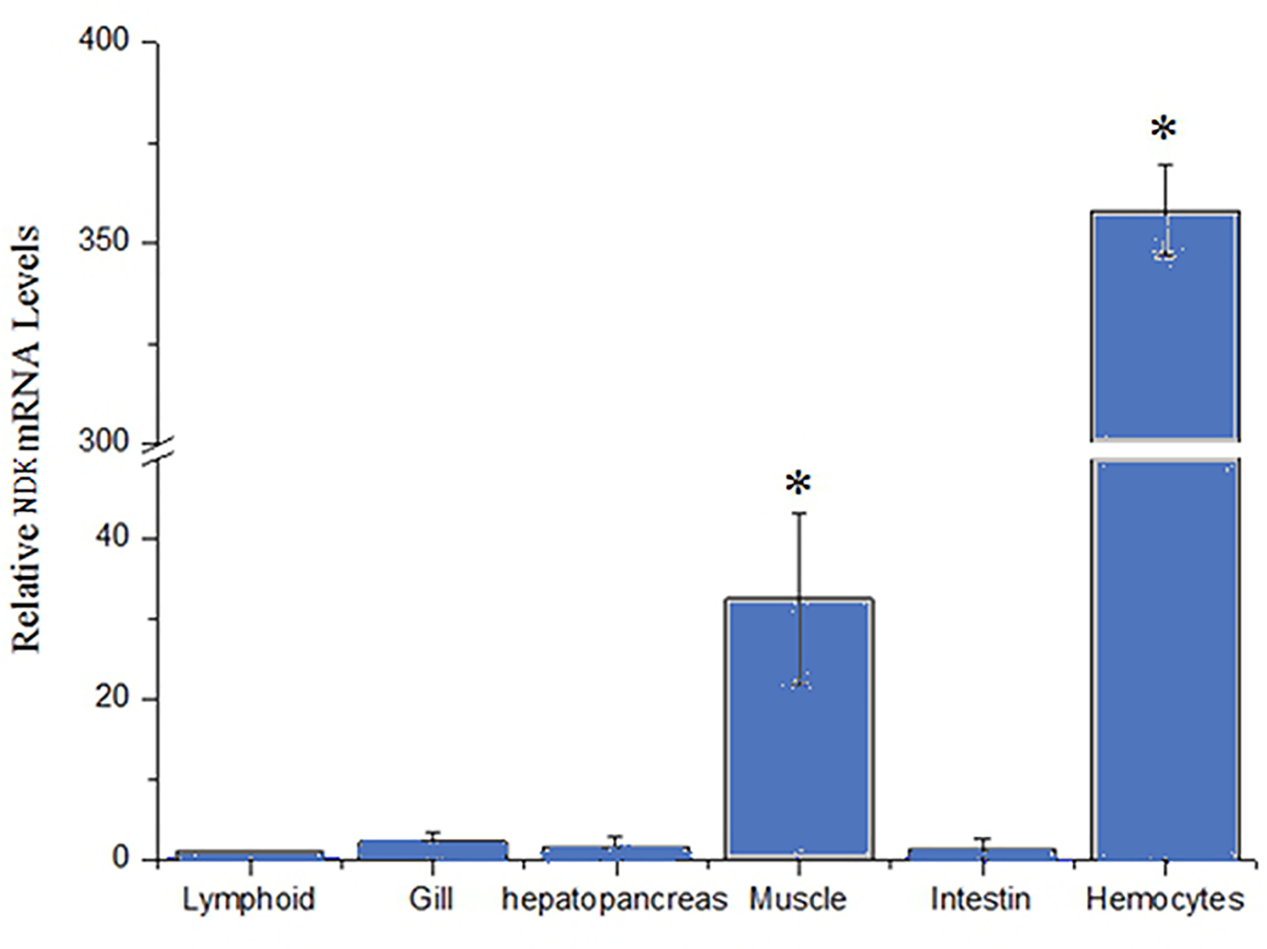
Relative NDK mRNA levels in shrimps tissues. Relative NDK mRNA levels in tissues of lymphoid, gill, hepatopancreas, muscle, intestine and hemocytes of experimentally shrimp *L*. *vannamei*. Bars represent the means±SE. Differences were evaluated with one-way ANOVA and Tukey test (p < 0.05).

We discovered different levels of NDK expression in the six tissues (Fig 2). In hemocytes and muscle, gene expression of NDK was significant higher than other tissues, especially in hemocytes. Hemocytes play an essential role in physiology and immune defense of shrimp [21], so results of NDK mRNA levels indicated that NDK could have some important functions in shrimps.

### WSSV copy number quantification by qPCR

**Fig 3:**
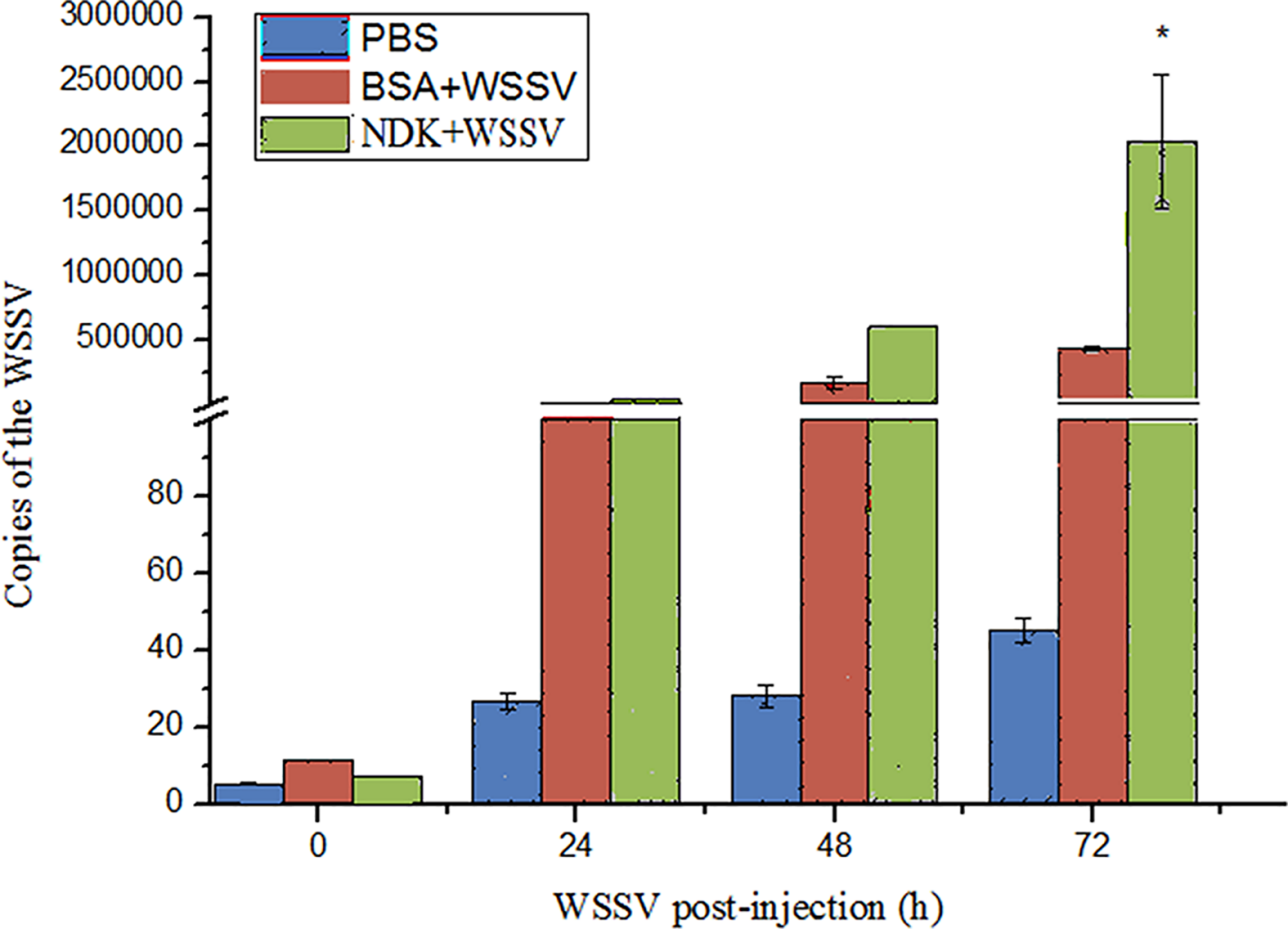
WSSV copies number. Estimated WSSV copies number/ng of DNA in gill of experimentally infected shrimp *L*. *vannamei* among NDK group, BSA group and the control group from a time course study.

The results of qRT-PCR indicate that WSSV copies increased significantly (P < 0.05) in WSSV-infected group which injected into rNDK by comparison to the BSA and PBS groups. (Fig 3). At 72 h post virus inoculation, WSSV copy number in the rNDK+WSSV group was higher (2.0×10^6^ copies/ng) than the BSA+WSSV group (≈ 4.5×10^5^ copies/ng). These results suggest that rNDK contributed to increase WSSV infectivity

### Inhibition of NDK gene expression in shrimp by dsRNA-NDK

**Fig 4:**
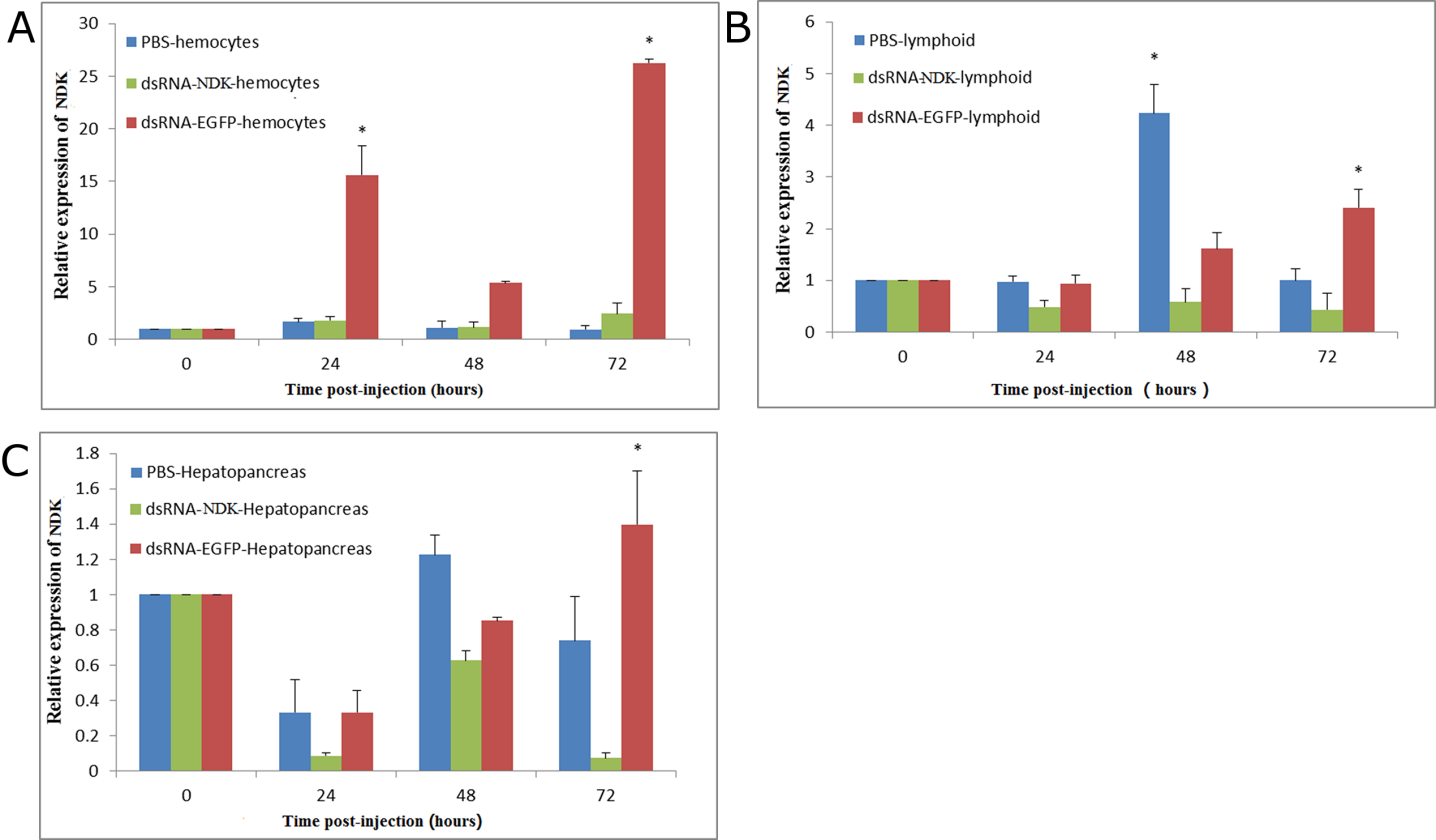
Temporal expression of NDK in three tissues. (A) Temporal expression of NDK in hemocytes, (B) Temporal expression of NDK in lymphoid (C) Temporal expression of NDK in hepatopancreas. Healthy *L*. *vannamei* were injected intramuscularly at the third abdominal segment with 20μL of PBS (control group), 1μg/g of dsRNA-NDK and dRNA-EGFP. At 0, 3, 6, 12, 24, 36, 48 and 72 h, three shrimp were randomly selected from each group from which the hemocytes, lymphoid and hepatopancreas for qPCR analysis. hemocytes, lymphoid and hepatopancreas

The results showed that the gene expression of NDK in hemocytes, lymphoid and hepatopancreas was significantly lower from 0 h to 72 h compared to PBS and dsRNA-EGFP groups. Therefore, it was shown that the NDK gene was inhibited by dsRNA-NDK (*P* < 0.05) (Fig 4A–4C). Otherwise, the level of NDK in shrimp hemocytes from the dsRNA-EGFP group was significantly higher than the two other groups. The reason of this result is not clear

### Relative quantification of *IE1* and *VP28* gene expression in gills of WSSV-infected shrimp

**Fig 5:**
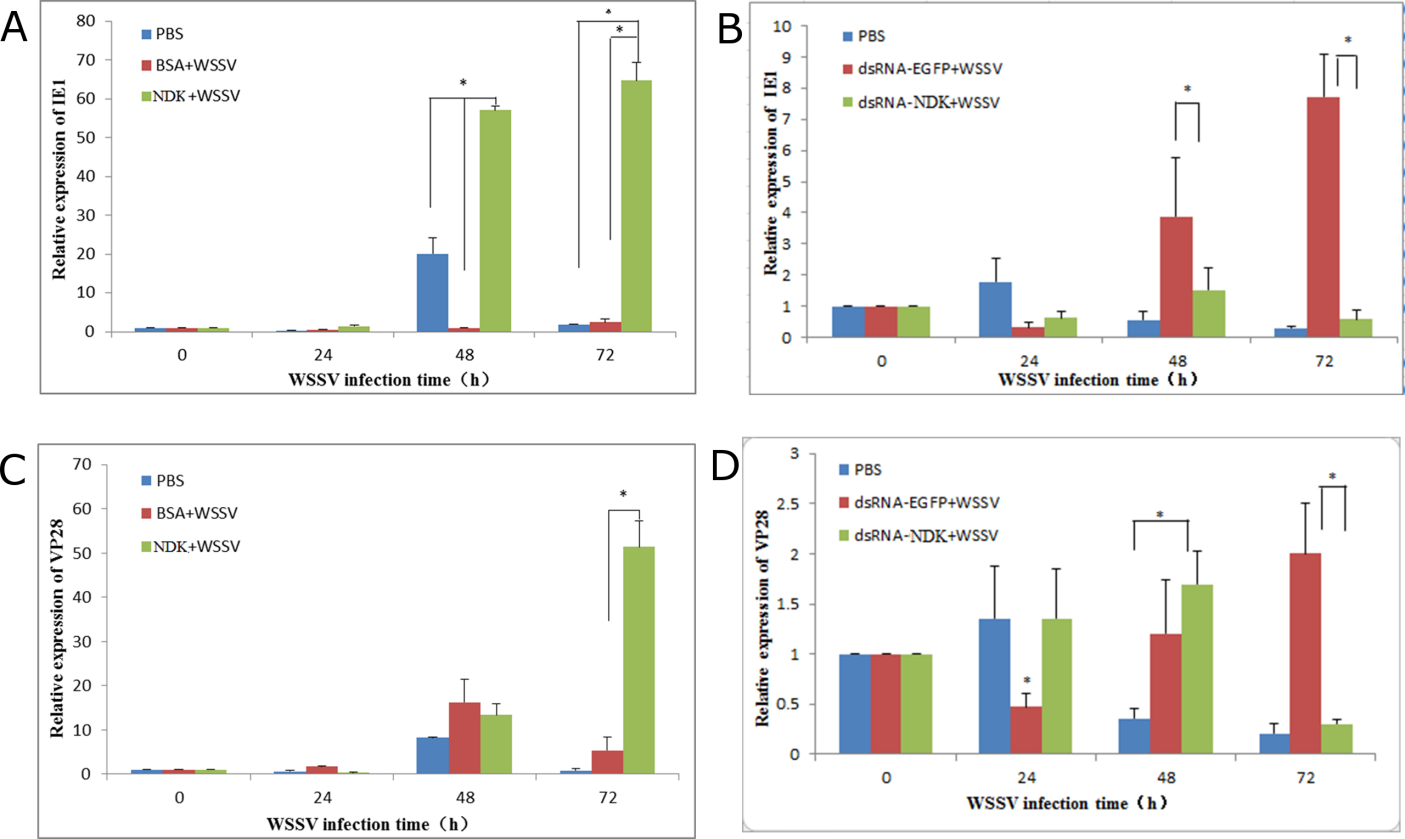
Relative IE1 and VP28 mRNA levels in experimental groups. (A) Relative IE1 mRNA levels of WSSV infected shrimp including over-expressed NDK group, BSA group and PBS group. (B) Relative IE1 mRNA levels of WSSV infected shrimp including dsRNA-NDK group, dsRNA-EGFP group and PBS group. (C) Relative VP28 mRNA levels of WSSV infected shrimp including over-expressed NDK group and BSA group. (D) Relative VP28 mRNA levels of WSSV infected shrimp including dsRNA-NDK group, dsRNA-EGFP group and PBS group. Data represent the means ± SE. Differences were evaluated with one-way ANOVA and Tukey test (p < 0.05).

After WSSV infection, the gene expression of *IE1* and *VP28* changed with time. The *IE1* gene in gills was detected to be up-regulated from 48 h to 72 h during WSSV infection in the NDK group. Significant differences in *IE1* gene expression existed between NDK and the control groups BSA and PBS during this time (*P* < 0.05; Fig 5A). Meanwhile, we found that the level of *IE1* gene in PBS group was significantly higher than BSA group at 48 h. However, the gene expression of *VP28* in NDK infected group reached their highest level at 72 h and there were no significant changes observed from 24 h to 72 h in the BSA infected group and non-infected control group (*P* < 0.05; Fig 5C).

Furthermore, the gene expression of *IE1* (Fig 5B) in the dsRNA-NDK infected group and PBS group was significantly lower than dsRNA-EGFP infected group at 48 h and 72 h. But the level of *VP28* gene (Fig 5D) in the dsRNA-NDK infected group was significantly higher than in the dsRNA-EGFP group and PBS group at 48 h. An effective inhibition of NDK was achieved by dsRNA-NDK at 72 h compared to the dsRNA-EGFP group. In contrast, in the dsRNA-EGFP group, gene expression of IE1 and VP28 was up-regulated from 48 h to 72 h.

From the results we conclude that upon WSSV infection, gene expression of IE1 at 72 h and VP28 at 48 h and 72 h increased in shrimps, whereas treatment with dsRNA-NDK contributed to suppress WSSV genes IE1 and VP28 expression in gills.

### Effect of expressed NDK and dsRNA-NDK on the cumulative mortality of WSSV-infected shrimp

**Fig 6:**
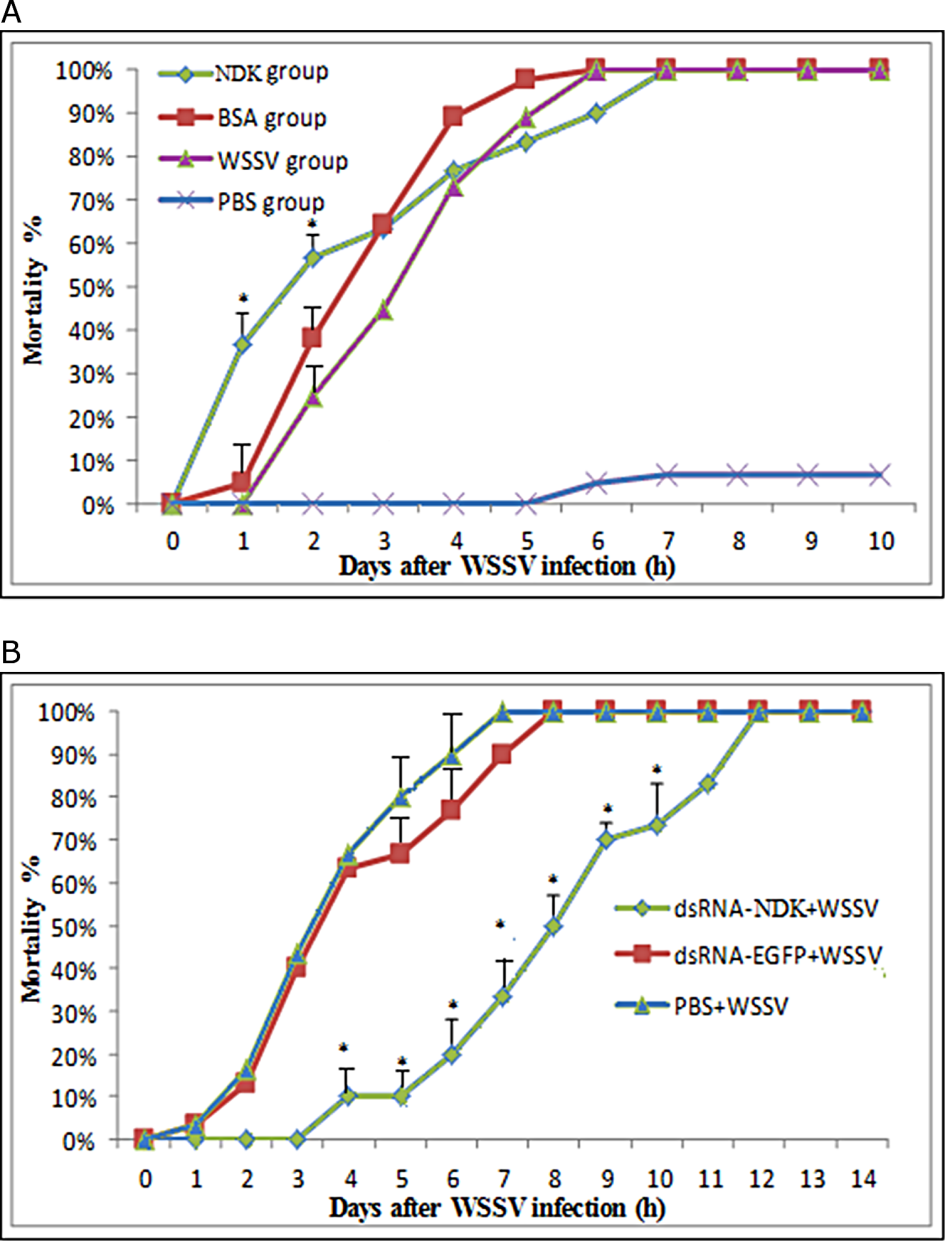
Time-mortality relationships of over-expressed NDK and followed by WSSV challenge. (A) Cumulative mortality of shrimps administrated with NDK, BSA, WSSV and PBS. Mortality was measured in each treatment group (n = 45) and was daily recorded. Analyses with SPSS 18.0 tested differences in mortality between groups (NDK, BSA and WSSV). Significant differences in mortality were only found in days 1 and 2 (P < 0.05). (B) Mortality of WSSV-infected shrimps treated with dsRNA-NDK was delayed for 4 days compared to dsRNA-EGFP- and negative control groups. The abscissa indicates time (days post WSSV challenge) and the ordinate indicates cumulative mortality (%). Significant differences in mortality were found between dsRNA-NDK and the other groups since day 2 post WSSV challenge (P < 0.05).

To further evaluate the relationship between rNDK and WSSV, we designed two experiments to be carried out in vivo. In the first experiment, at 6–7 days post WSSV inoculation all the shrimps in the WSSV-infected groups had died but variations in time to mortality were observed. At 1–2 day post WSSV inoculation, mortality in the WSSV-NDK group was significantly higher than the other groups (P < 0.05) (Fig 6A). Nonetheless, all the groups showed 100% mortality between 5–7 days post WSSV inoculation. It is possible that NDK might play a role in promoting WSSV replication early during infection.

In the other experiment, WSSV combined with dsRNA-NDK was used to check the function of rNDK in WSSV-infected shrimps. The data showed that the cumulative mortality of WSSV-infected shrimps was significantly delayed by dsRNA-NDK (P < 0.05) (Fig 6B) compared to the WSSV-challenged groups. Mortality (100%) was delayed 4 days in this group compared to the other groups. As a result of this experiment, we further concluded that the function of rNDK was to promote virus replicationv at the early stages of WSSV infection.

## Discussion

NDK is a key enzyme in controlling cell energy and nucleotide metabolism, and could act as a chemotherapeutic prodrug in some pathologies since it functions in DNA replication and viral infections [22]. However, there are few studies that show the immune response of NDK to WSSV in *L*. *vannamei*. Accordingly, we used a prokaryotic expression method to acquire rNDK to estimate the specificity of NDK binding to WSSV and to investigate if NDK would function in preventing WSSV in cultured shrimp.

RNAi has recently been observed as a common mechanism for post-transcriptional gene silencing in a variety of eukaryotic organisms, and is widely proven as an effective means to suppress viral infection or the replication of many viruses [12–14]. Therefore, the synthesis of double-stranded RNA (dsRNA-NDK oncoprotein) was used to investigate the role of NDK in WSSV infection in shrimps. Using a similar method, we hope to investigate what role NDK plays in WSSV-infected shrimps.

Firstly, tissues distribution analysis indicated that gene expression of NDK was higher in hemocytes than in other shrimp tissues. However, gills were a main tissue extracted in our experiment because they play an important role in defense, whereas hemocytes have been the main cells studied so far [23, 24], followed by hepatopancreas [25] and lymphoid tissue, in research related to specialized defense in invertebrates [26]. In Fig 3, we observed that WSSV copy number in the NDK+WSSV infection group was higher than in the BSA+WSSV and PBS groups, especially at 72 h post WSSV inoculation. This result suggests that NDK might promote WSSV replication early during infection in shrimps.

Otherwise, results of NDK gene expression in shrimps injected with dsRNA-NDK showed that synthesized dsRNA-NDK had a significant function in inhibiting expression of the NDK gene. Subsequently we analyzed relative expression of *IE1* and *VP28* in gills of WSSV-infected shrimp. A previous study indicated that the WSSV immediate early gene *IE1* is highly expressed throughout the viral infection cycle and may play a central role in initiating viral replication during infection [27]. In our study, *VP28* and *IE1* were measured by qPCR to identify any variation in their expression. When shrimps were injected with rNDK protein, we found that gene expression of *IE1* increased significantly at 48 h and 72 h compared to the PBS group and BSA+WSSV infection group. In contrast, in the dsRNA-NDK group, NDK levels were lower at 48 h and 72 h post WSSV inoculation than in the dsRNA-EGFP+WSSV and PBS groups, upon WSSV challenge. A similar trend in VP28 gene expression was found only at 72 h post WSSV inoculation. We concluded that upregulation of IE1 and VP28 gene expression was due to the presence of rNDK protein.

In order to further investigate the phenomenon, we analyzed results of cumulative mortality to show that mortality in the NDK+WSSV group was significantly higher than other WSSV-infected groups from 1 to 2 days. Furthermore, in the dsRNA-NDK group, large amounts of shrimps were alive at the early stage of infection. So, we suggested that rNDK protein could increase mortality of WSSV-infected shrimps and dsRNA-based NDK molecules could provide significant and long-term protection in this pathology.

As few studies were carried out to prove what roles NDK acted in *L*. *vannamei* infected by WSSV, no reference could be used. Therefore, combined with the results obtained based on the limited shrimps, we concluded that dsRNA-NDK had a modest antiviral effect at the early stages of WSSV infection in shrimps. Though protective effect was not persistent, it could also act as potential therapy for WSSV-infected shrimps. However, some other studies have indicated that NDK-1 is necessary for proper MAPK activation in the somatic tissues of the worm [28]. In order to further understand the function of NDK, we need to research its function in cell metabolism in the shrimp.

In summary, these results indicate that NDK is a protein which could influence the adverse function of WSSV in shrimp. However, more studies should be carried out to further support this research because the mechanism of how NDK acts on WSSV is not proven sufficiently. Nevertheless, our research also leads the way to further study the specific function of NDK in WSSV, providing a basis for addressing this issue in the near future.

## Conclusions

In our study, recombinant protein NDK was produced to study the mechanisms regulating WSSV in *L*. *vannamei* by qRT-PCR, and RNAi, combined with analyzed data of *IE1* and *VP28* gene expression. We found that recombinant protein NDK could induce rapid infection of WSSV and accelerate the death of shrimps. Therefore, to inhibit the expression of NDK in *L*. *vannamei* might strengthen immune defenses against infection by the pathogen WSSV.
